# Damage control management of innominate artery injury with tracheostomy

**DOI:** 10.1186/s40792-016-0144-7

**Published:** 2016-02-16

**Authors:** Yohei Okada, Hiromichi Narumiya, Wataru Ishii, Iiduka Ryoji

**Affiliations:** Department of Emergency and Critical Care Medicine, Japanese Red Cross Kyoto Daini Hospital, 355-5 Haruobi-cho, Kamigyo-ku, Kyoto, 602-8026 Japan

**Keywords:** Damage control surgery, rSO_2_, Near-infrared spectroscopy, Tracheo-innominate fistula, Innominate artery

## Abstract

Innominate artery injury is a rare, but catastrophic complication of tracheostomy. We present a case of severe hemorrhagic shock in a 79-year-old male with innominate artery injury that occurred during tracheostomy. Despite temporary innominate artery isolation, the regional forehead saturation was 60 % without laterality. Because adequate cerebral blood flow was apparently maintained through collateral flow, we ligated the innominate, right carotid, and subclavian arteries. We confirmed adequate blood flow to the brain and the right subclavian artery through collateral circulation after ligation using computed tomographic angiography. A damage control management, which involves ligating the injured innominate artery to arrest hemorrhage and monitoring regional forehead saturation for brain ischemia, can be a considerable procedure for the treatment of severe hemorrhagic shock due to innominate artery injury.

## Background

Innominate artery (IA) injury, including formation of a tracheo-innominate fistula, is a rare but severe complication of tracheostomy that is associated with high mortality due to the resultant severe hemorrhagic shock [1]. A damage control strategy consisting of prompt control of bleeding and management to maintain physiological homeostasis—crucial to avoiding the deadly triad of hypothermia, acidosis, and coagulopathy—is essential in a patient with severe hemorrhagic shock, such as following a neck vessel injury [2]. Moreover, generally, the neck vessel surgery has the risk of severe neurological complications, so that we should avoid brain ischemia as possible in such a case.

## Case presentation

A 79-year-old male, with terminal lung cancer at the right upper lobe, had been in another hospital and intubated for 3 weeks since an accidental airway obstruction. His doctor performed the tracheostomy in a surgical manner at the bedside. During the procedure, continuous arterial bleeding at the surgical site occurred. The doctor could not detect the bleeding artery clearly, but direct manual compression with an index finger at the surgical site provided temporary hemostasis. On arrival at our emergency department, he was intubated and in severe hemorrhagic shock despite having received transfusion of six units of packed red blood cells concentration (PRBC) and six units of fresh frozen plasma (FFP).

Arterial blood gas assessment revealed metabolic and respiratory acidosis and anemia [pH 7.09, pCO_2_ 71 mmHg, pO_2_ 193 mmHg, HCO_3_^−^ 21.0 mmol/L, base excess (BE) −8.3 mmol/L, lactate (Lac) 5.5 mmol/L, hemoglobin (Hb) 7.9 g/dL, hematocrit (Ht) 22 %, FiO_2_ 1.0], and blood tests showed coagulopathy [PT 35.3 %, PT-INR 1.68, activated partial thromboplastin time (APTT) 56.2 s, fibrinogen (Fib) 84 mg/dL]. These data showed that his condition was in severe hemorrhagic shock and coagulopathy.

The patient was taken up for emergency surgery in view of the serious nature of the injury.

**Fig. 1:**
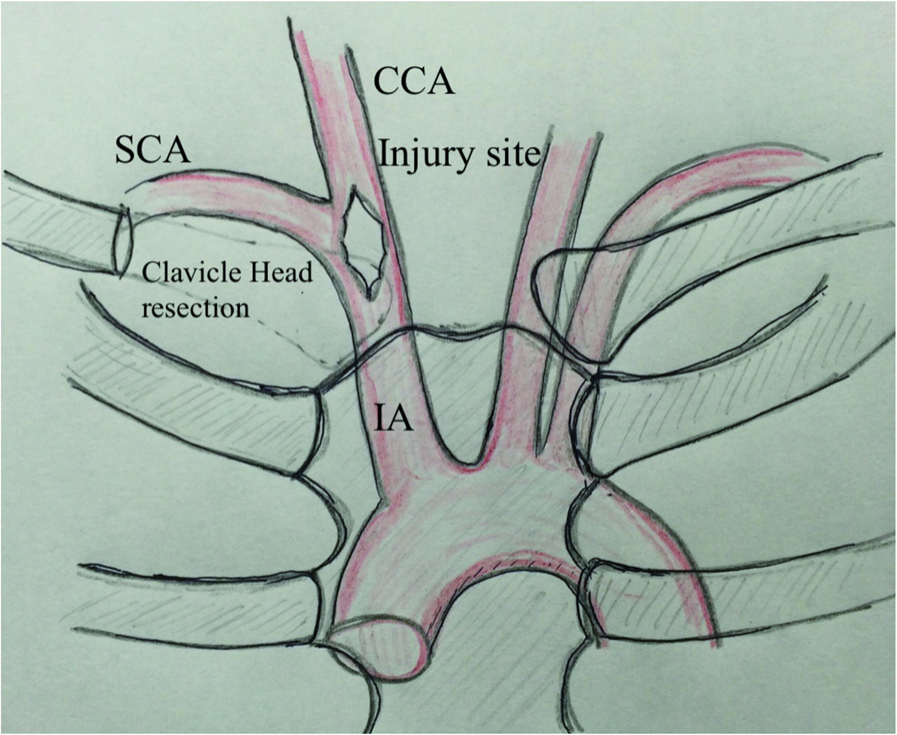
Diagram of the innominate arterial injury and the surgical approach. A right supraclavicular incision with resection of the right clavicle head revealed injury to both innominate and right common carotid arteries. *IA* innominate artery, *SCA* subclavian artery, *CCA* common carotid artery

During surgery, because of the clinical impression of an injury to the distal IA or proximal common carotid artery, a right supraclavicular incision was made, followed by dissection of the platysma, division of the right sternocleidomastoid muscle, and resection of the right clavicular head. The exposure revealed that both innominate and right common carotid arteries were injured (Fig. 1). After innominate, the right subclavian and right common arteries were isolated and primary repair and graft interposition of the arteries was attempted, but proved too difficult because of atherosclerosis. After an hour into the surgery, the patient showed worsening hypothermia (34 °C), metabolic acidosis, and clinical evidence of coagulopathy; therefore, we decided a damage control strategy. Despite clamping of the IA for about 10 min in attempting primary repair, the regional oxygen saturation (rSO_2_) of the forehead, measured by near-infrared spectroscopy (NIRS; INVOS 5100C Cerebral/Somatic Oximetry Monitors™; Covidien, USA), was approximately 60 % without laterality. It seemed that adequate blood circulation to the brain was maintained through the collateral flow; therefore, we decided to ligate the innominate, right carotid, and subclavian arteries. The wound was packed with gauze for temporary wound closure. Overall, the operation lasted for 1 h and 25 min, with a recorded blood loss of 1190 g and transfusion of 16 units PRBCs and 10 units of FFP.

**Fig. 2:**
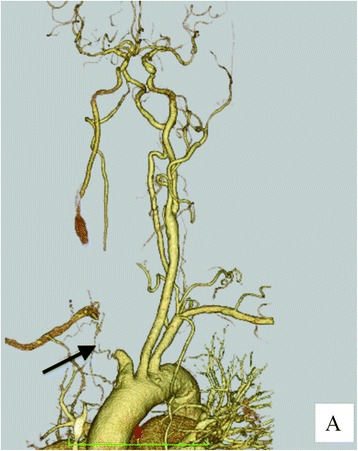
CT angiography of neck vessels. (*Arrow*) Ligation of innominate artery**.** The distal right common carotid and right subclavian arteries were enhanced by collateral flow through the left common carotid artery and the circle of Willis

Postoperative contrast-enhanced computed tomography revealed no apparent acute cerebral ischemia and extravasation, and the distal right common carotid and right subclavian arteries were enhanced through the circle of Willis (Fig. 2). A right tracheal shift due to adhesions caused by lung cancer was also revealed, indicating a course of the IA anterior to the third tracheal ring.

After admission to the intensive care unit, coagulopathy and hypothermia were corrected with massive transfusion (overall transfusions: PRBCs 24 units and FFP 26 units).

No symptoms of hypo-perfusion of the right upper limbs and brain were observed; therefore, an extra-anatomical bypass was deemed unnecessary. On postoperative day 1, we closed the wound definitively. On postoperative day 7, the patient was transferred to a sub-acute, community hospital.

## Discussion

IA injury is a rare, but severe, complication of tracheostomy that is associated with high mortality due to the resultant severe hemorrhagic shock [1]. Such an injury may be the result of an anatomical anomaly [3]. In the present case, a right tracheal shift due to adhesions caused by lung cancer and the course of the IA anterior to the third tracheal ring were the major causative factors for the catastrophe. Endovascular management became the method of choice when treating a stable patient with an IA injury, but should be avoided in a hemodynamically unstable patient, who would require expeditious transfer to the operating room [4].

The most preferred exposure for a penetrating ZONE 1 neck injury (from the bottom of the cricoid cartilage to the clavicles) is a median sternotomy combined with an anterior neck or supraclavicular incision. Another approach involves a supraclavicular incision with or without resection of the head of the clavicle [5]. However, in this case, we selected a supraclavicular incision with clavicular head resection to obtain adequate exposure, while also preparing for a median sternotomy, because we thought median sternotomy had hemorrhagic risk in view of possible adhesions due to lung cancer.

Surgical procedures are divided into two strategies: one is a ‘definitive repair’ including primary repair as direct suture, graft interposition, and a bypass graft from the ascending aorta, and the other is a ‘damage control strategy’. The concept of a damage control strategy has immediate control of bleeding and temporary wound closure, as its objective minimized operative time and intervention for at a moment of physiological frailty, and planed re-surgery [6]. In general, direct suture and graft interposition with vein patch or pericardium are the first choice of treatment [5] but were too difficult to carry out in the present case because of the patient’s atherosclerosis. Bypass grafting from the ascending aorta has been reported as a radical treatment in some cases [7]. However, this patient showed physiological frailty, which was worsening hypothermia (34 °C), metabolic acidosis, and clinical evidence of coagulopathy also known as the ‘deadly triad’; therefore, we had to choose a balloon tamponade, temporary shunt, or ligation as the damage control method [8]. A balloon tamponade is one of considerable damage control methods, but we could not prepare it at the moment. A temporary shunt is a damage control method for a vascular injury and is more commonly associated with limb fracture. It involves the insertion of a plastic tube into the injured vessel that is then tightly secured in place with a silk tie to ensure that the tube will not be dislodged [8]. Complications of this procedure include re-bleeding and thrombus embolization. In the present case, we were wary of generating an embolism to the brain; therefore, we tend to avoid these methods. However, if the injury were to the subclavian artery or another vessel, excepting the common carotid artery, we would have used this procedure as a damage control method. Ligation is the easiest and fastest method to control bleeding but is associated with the risk of ischemia and neurologic deficit. In some previous articles reporting on innominate artery injury caused by a trachea-innominate fistula, ligation of the innominate artery could be considered as the first choice to control massive hemorrhage and was associated with a high survival rate with regard to re-bleeding and infection [9]. Previous articles have reported that such flow interruption would not yield significant neurologic or vascular complications because of the collateral flow through the left common carotid artery and the circle of Willis [9].

Monitoring rSO_2_ by near-infrared spectroscopy (NIRS) is an invasive method for detecting brain ischemia in circumstances such as carotid surgery [10]. In a previous article [11], it was reported to be useful in deciding the surgical management of bleeding from a bracheo-innominate fistula. In the present case, after isolation of the innominate artery, the patient’s rSO_2_ values seemed adequate to obtain adequate blood flow to the brain without laterality; therefore, we decided to proceed with the ligation of the innominate artery immediately. On monitoring after ligation, we noted that the value had not changed. Monitoring the rSO_2_ is considered as a useful method to determine selection of the surgical procedure. In our case, we confirmed adequate blood flow to the brain and the right subclavian artery through collateral circulation after ligation using computed tomographic angiography. In a damage control strategy, planed re-surgery is usually performed. However, this case showed no symptoms of hypo-perfusion of the right upper limbs and brain after ligation; therefore, we did not perform planed re-surgery as an extra-anatomical bypass.

## Conclusions

As a damage control strategy, ligation of the IA together with monitoring of the rSO_2_ of the forehead for evaluating brain ischemia can be considered a preferable procedure for the treatment of severe hemorrhagic shock due to IA injury.

## Consent

Written informed consent was obtained from the patient for publication of this case report and any accompanying images. A copy of the written consent is available for review by the editor of this journal.
